# Partial anomalous pulmonary venous connection associated with hemoptysis: A case report

**DOI:** 10.1097/MD.0000000000015893

**Published:** 2019-06-07

**Authors:** ChengCheng Li, Peng Teng, Yanyan Yang, Yiming Ni, Liang Ma

**Affiliations:** a Department of Cardiothoracic Surgery; b Department of Pharmacy, The First Affiliated Hospital, College of Medicine, Zhejiang University, Zhejiang Province, P.R. China.

**Keywords:** congenital, hemoptysis, lobectomy, partial anomalous pulmonary venous connection

## Abstract

**Rationale:**

Partial anomalous pulmonary venous connection (PAPVC) is a rare congenital anomaly characterized by the failure of fusion of embryologic pulmonary venous system with left atrium.

**Patient Concerns:**

A 45-year-old male patient with PAPVC who was hospitalized because of mild hemoptysis. Images showed the anomalous vein originated from the left upper pulmonary vein and flowed into the left brachiocephalic vein. No other underlying causes for hemoptysis were detected.

**Diagnosis:**

After multi-disciplinary discussion, the patient was diagnosed as PAPVC of left upper pulmonary vein draining into the left brachiocephalic vein with intact atrial septum.

**Interventions:**

Although surgical correction of PAPVC was feasible, left upper lobectomy was performed as the definitive treatment for both hemoptysis and PAPVC.

**Outcomes:**

The patient had an uneventful postoperative hospital course and was followed up for nearly 2 years without recurrence of hemoptysis.

**Lessons:**

PAPVC is associated with atrial septal defect in 80% to 90% of cases while isolated PAPVC with intact atrial septum is an extremely rare entity. We present a rare isolated PAPVC patient with hemoptysis. To our best knowledge, PAPVC associated with hemoptysis has never been reported before.

## 1 Introduction

Partial anomalous pulmonary venous connection (PAPVC) is a rare congenital anomaly characterized by the failure of fusion of embryologic pulmonary venous system with the left atrium, which manifested as 1 or more, but not all, of the pulmonary veins draining into the right atrium, coronary sinus or a systemic vein.^[1]^ Its prevalence is approximately 0.4% to 0.7% in the general population, with 10% of cases being left-sided and 80% to 90% of cases being associated with atrial septal defect (ASD).^[2,3]^ PAPVC creates a left-to-right shunt which is responsible for the associated symptomatology. Most patients with PAPVC are asymptomatic while a few patients present symptoms secondary to right-sided fluid overload, pulmonary vascular disease, and worsening right-sided heart failure. The common symptoms of PAPVC include dyspnea, decreased exertional tolerance and peripheral edema.^[4]^ Herein, we report a rare case of PAPVC of left upper pulmonary vein draining into the left brachiocephalic vein with intact atrial septum, which presented with hemoptysis and was finally treated with lobectomy. To our best knowledge, PAPVC associated with hemoptysis has never been reported before.

## 2 Case presentation

**Figure 1:**
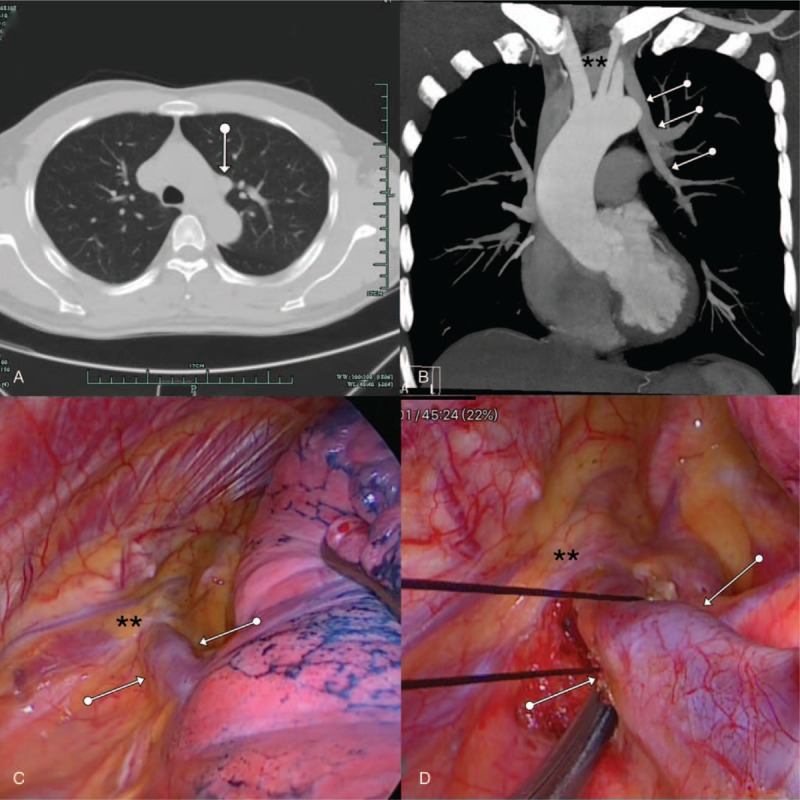
(A) An anomalous vessel were found near the aortic arch while no malignant tumor, tuberculosis, pneumonia, or bronchiectasis were detected by chest CT; (B) Contrast-enhanced chest CT revealed that the left upper pulmonary vein (white arrows) drained into the left brachiocephalic vein (asterisks); (C, D) Intraoperatively, the left upper pulmonary vein (white arrows) was confirmed draining into the left brachiocephalic vein (asterisks). CT = computed tomography.

A 45-year-old Chinese male, without any positive medical and family history, was admitted for intermittent mild hemoptysis (<30 mL/24 hour) for previous 2 weeks, with no other complaints. Physical examination was unremarkable. Laboratory test revealed negative T-SPOT.TB test and the normal inflammatory makers including white blood cells, high-sensitivity C-reactive protein, and procalcitonin. Enlarged mediastinal silhouette was found on the chest roentgenogram. Transthoracic echocardiography (TTE) suggested the possibility of PAPVC involving left upper pulmonary vein. No ASD, patent foramen ovale or other cardiac anomalies were detected. In addition, TTE revealed mild tricuspid regurgitation with pulmonary artery systolic pressure estimated of 35mmHg. Further assessment by contrast-enhanced computed tomography (CT) showed the anomalous left upper pulmonary vein draining into the left brachiocephalic vein (Fig. 1A and B). No occupying lesion, pneumonia, tuberculosis, bronchiectasis, and arteriovenous malformation were found on the contrast-enhanced CT. In addition, hematocele was detected on the opening of the left upper lung bronchus while no bronchial tumor was detected by bronchoscopy. According to extensive workup, the common causes of hemoptysis, including malignant tumor, tuberculosis, pneumonia, fungal infections, bronchiectasis, and bronchitis, were excluded. A multi-disciplinary meeting was held and concluded that patient may not benefit from a PAPVC repair because hemoptysis remission could not be guaranteed. After full communication with patient, the decision of left upper lobectomy was finally made as definitive treatment for both hemoptysis and PAPVC.

Video-assisted thoracoscopic the left upper lobectomy was performed under one-lung ventilation. The anomalous vein originated from the root of left upper pulmonary vein, traversed aortic arch and connected to the left brachiocephalic vein (Fig. 1C and D). Left upper lobectomy with ligation of the anomalous vein was performed successfully. The patient had an uneventful recovery without any complications and was discharged home on the 5th postoperative day. The patient has been followed up for almost 2 years without recurrence of hemoptysis.

## 3 Discussion

PAPVC is a rare congenital anomaly with a reported incidence of 0.4% to 0.7%.^[2]^ It is frequently right-sided in about 90% cases and associated with ASD in 80% to 90% cases,^[3,5]^ which makes our case very uncommon as it was a left-sided isolated lesion. The left-to-right shunt caused by PAPVC may result in high output state and significant right heart volume overload, which leads to progressive dilation of both tricuspid annulus and right ventricle. The symptoms are mainly dependent on the severity of the shunt. A large shunt, assessed by pulmonary-to-systemic blood flow ratio (Qp/Qs), can cause right heart failure and pulmonary hypertension with increased pulmonary vascular resistance.^[6]^ The majority of the adult patients diagnosed with PAPVC presents with fatigue, dyspnea, palpitations, chest pain or peripheral edema.

The definitive treatment of PAPVC is surgical separation of the pulmonary venous system from the systemic venous system through redirection of anomalous pulmonary vein(s) into the left atrium. Most centers and literatures do not support intervene for Qp/Qs <2.0 and isolated PAPVC.^[7]^ However, there is still no consensus on the timing of surgical intervention in PAPVC when other lung issues are involved, because of the complexity and the risk, including pulmonary vein stenosis and residual shunt, of the procedure. Lobectomy may also be considered when PAPVCs are encountered in the patients with other lung issues like tumor and bronchiectasis.

Hemoptysis is the expectoration of blood to mouth or nose that originates from the respiratory tract. Hemoptysis can originate from various diseases such as acute benign disease (including bronchitis), chronic benign disease (including bronchiectasis, tuberculosis), or malignant tumor. Contrast-enhanced chest CT is highly recommended and provides valuable information for the etiologies of hemoptysis.^[8]^ Cryptogenic hemoptysis, defined as cases in which both nondiagnostic, makes up approximately 10% to 20% of hemoptysis cases.^[8]^

In our case, our patient had PAPVC of left upper pulmonary vein draining into the left brachiocephalic vein and presented with mild hemoptysis. Contrast-enhanced CT, T-SPOT.TB, and even bronchoscopy were performed and the common causes of hemoptysis like malignant tumor, bronchiectasis, tuberculosis, and arteriovenous malformation were excluded. Based on the bronchoscopic findings of hematocele on the opening of the left upper lung bronchus, hemoptysis was considered highly correlated with PAPVC, which was extremely rare. To our best knowledge, PAPVC presented with hemoptysis has never been reported before. Although surgical repair of PAPVC is effective, we could not ensure the remission of the hemoptysis after surgery because of its unknown underlying mechanisms. In addition, due to the higher pressure of left atrium than the left brachiocephalic vein and the possibility of pulmonary vein stenosis, surgical repair may cause increased pulmonary venous pressure and subsequent pulmonary congestion, which would aggravate the hemoptysis. Based on above consideration, we finally performed left upper lobectomy as the definitive treatment for both PAPVC and hemoptysis.

## 4 Conclusion

We experienced a patient with PAPVC associated with hemoptysis, which is extremely rare. Contrast-enhanced CT is of great importance and value for evaluation of both PAPVC and hemoptysis. Management of both entities is also an uncommon clinical scenario that requires careful evaluation and consideration. For patients in whom PAPVC and hemoptysis are considered highly correlated, lobectomy is appropriate.

## Author contributions

**Conceptualization:** Peng Teng.

**Data curation:** Yanyan Yang.

**Methodology:** ChengCheng Li, Liang Ma.

**Supervision:** Yiming Ni.

**Writing – original draft:** Peng Teng.

**Writing – review & editing:** ChengCheng Li.

## References

[1] Majdalany DS Phillips SD Dearani JA Isolated partial anomalous pulmonary venous connections in adults: twenty-year experience. Congenit Heart Dis 2010;5:537–45. 21106012 10.1111/j.1747-0803.2010.00458.x

[2] Mikubo M Ikeda S Hoshino T Pulmonary resection of lung cancer in a patient with partial anomalous pulmonary venous connection. Ann Thorac Surg 2013;95:1799–801. 23608268 10.1016/j.athoracsur.2012.10.033

[3] Javangula K Cole J Cross M An unusual manifestation of left partial anomalous pulmonary venous connection. Interact Cardiovasc Thorac Surg 2010;11:846–7. 20805252 10.1510/icvts.2009.231100

[4] Edwin F Left-sided partial anomalous pulmonary venous connection—should diagnosis lead to surgery? Interact Cardiovasc Thorac Surg 2010;11:847–8. 21097462 10.1510/icvts.2009.231100A

[5] Zhang Z Zhang L Xie F Echocardiographic diagnosis of anomalous pulmonary venous connections; Experience of 84 cases from 1 medical center. Medicine (Baltimore) 2016;95:e5389. 27858923 10.1097/MD.0000000000005389 PMC5591171

[6] Babb JD McGlynn TJ Pierce WS Isolated partial anomalous venous connection a congenital defect with late and serious complications. Ann Thorac Surg 1981;31:540–1. 7247546 10.1016/s0003-4975(10)61345-8

[7] Hijii T Fukushige J Hara T Diagnosis and management of partial anomalous pulmonary venous connection. A review of 28 pediatric cases. Cardiology 1998;89:148–51. 9524017 10.1159/000006771

[8] Ketai LH Mohammed TL Kirsch J ACR appropriateness criteria( hemoptysis. J Thorac Imaging 2014;29:W19–22. 24717602 10.1097/RTI.0000000000000084

